# The Expression of Superoxide Dismutase (SOD) and a Putative ABC Transporter Permease Is Inversely Correlated during Biofilm Formation in *Listeria monocytogenes* 4b G

**DOI:** 10.1371/journal.pone.0048467

**Published:** 2012-10-31

**Authors:** Yujuan Suo, Yanyan Huang, Yanhong Liu, Chunlei Shi, Xianming Shi

**Affiliations:** 1 MOST-USDA Joint Research Center for Food Safety and Bor Luh Food Safety Center, School of Agriculture and Biology, Shanghai Jiao Tong University, Shanghai, China; 2 Molecular Characterization of Foodborne Pathogens Research Unit, Eastern Regional Research Center, Agricultural Research Service, U. S. Department of Agriculture, Wyndmoor, Pennsylvania, United States of America; Louisiana State University and A & M College, United States of America

## Abstract

Little is known about the molecular basis of biofilm formation in *Listeria monocytogenes*. The superoxide dismutase (SOD) of the deletion mutant of lm.G_1771 gene, which encodes for a putative ABC transporter permease, is highly expressed in biofilm. In this study, the *sod* gene deletion mutant *Δsod*, and double deletion mutant of the *sod* and lm. G_1771 genes *Δ*1771*Δsod* were used to investigate the role of SOD and its relationship to the expression of the putative ABC transporter permease in biofilm formation. Our results showed that the ability to form a biofilm was significantly reduced in the *Δsod* mutant and the *Δ*1771*Δsod* double mutant. Both *Δsod* and *Δ*1771*Δsod* mutants exhibited slow growth phenotypes and produced more reactive oxygen species (ROS). The growth was inhibited in the mutants by methyl viologen (MV, internal oxygen radical generator) treatment. In addition, the expression of one oxidation resistance gene (*kat*), two stress regulators encoding genes (*perR* and *sigB*), and one DNA repair gene (*recA*) were analyzed in both the wild-type *L. monocytogenes* 4b G and the deletion mutants by RT-qPCR. The expression levels of the four genes were increased in the deletion mutants when biofilms were formed. Taken together, our data indicated that SOD played an important role in biofilm formation through coping with the oxidant burden in deficient antioxidant defenses.

## Introduction

*Listeria monocytogenes* is a Gram-positive foodborne pathogen capable of causing listeriosis both in human and animals. It is estimated that 99% of the listeriosis cases are caused by contaminated food products [1]. *L. monocytogenes* is widely distributed in the environment and food processing equipment. It can form biofilms, survive on food processing equipment for several years, and subsequently disperse to contaminate food products [2], [3]. Biofilms are structured communities of microorganisms adhering to a surface, and may be encapsulated within a self-produced protective and adhesive matrix of extracellular polymeric substances (EPS) [4]. Bacteria in biofilms are more resistant to various environmental stresses such as desiccation, UV light, antimicrobials, and sanitizers [5], [6]. Therefore, biofilms that are difficult to eliminate completely impose major challenges to the food industry.

Although environmental factors such as temperature, pH and medium composition affect the formation of biofilms [7], [8], factors required for biofilm formation in *L. monocytogenes* are still largely unknown. It has been shown that biofilm formation is related to oxidative stress as a response to adapting changes in environmental conditions in a number of bacteria [9], [10], [11], [12]. Oxidative stress can be induced inside biofilms to affect the growth of the bacteria, and result in reactive oxygen species (ROS) production, including superoxide (O^2−^), hydrogen peroxide (H_2_O_2_), hydroxyl radical (OH·), peroxyl radical (ROO·) and singlet oxygen (^1^O_2_) [13], [14]. ROS are highly toxic and can damage nucleic acids, proteins, and cell membrane fatty acids [15].

To prevent damage caused by oxidative stress, cells possess defense systems to detoxify ROS. These defense systems include: (i) genes that scavenge reactive oxygen, such as superoxide dismutases (*sod*) and catalases (*kat*), (ii) stress regulators that regulate some antioxidant genes expression, such as *perR* and *sigB*, and (iii) DNA repair genes that repair damaged DNA, such as *recA*. The function of these genes has been studied in *L. monocytogenes*, and it was found that some genes (*sod*, *sigB* and *recA*) were induced in biofilm formation [16], [17], [18].

In our previous study, we found that the lm.G_1771 gene (encoding a putative ABC transporter permease) negatively regulated biofilm formation in *L. monocytogenes* 4b G [19]. Two-dimensional gel electrophoresis (2-DE) and microarray analysis revealed that superoxide dismutase (SOD) was up-regulated 2-fold in the *Δ*1771 mutant compared to the wild-type 4b G [20]. SOD can participate in cellular detoxification and protect organisms against superoxides by dismutating the superoxide radical anion O_2_^−^ to H_2_O_2_, which is transformed into H_2_O by cellular catalase (*kat*) [21]. SOD can be classified into different types according to its metal cofactors. There are three *sod* genes in *Escherichia coli*, biofilm formation is significantly reduced in a *sodC* (CuZnSOD)-deleted mutant of *E.coli* O157:H7 [22]; only one SOD (MnSOD) is present in *L. monocytogenes* [23]. Proteomic analysis showed that in *L. monocytogenes* SOD was up-regulated in biofilms versus planktonic cells [16]. Thus, we speculate that SOD plays an important role in the biofilm formation of *L. monocytogenes*.

To study the role of SOD and its relationship with the putative ABC transporter permease in biofilm formation, two deletion mutants (*Δsod* and *Δ*1771*Δsod*) were constructed, and the biofilm formation and oxidative stress resistance were investigated together with the *Δ*1771 mutant. In addition, the relative expression (RE) levels of four genes including one oxidation resistance gene (*kat)*, two stress regulators encoding genes (*perR* and *sigB*), and one DNA repair gene (*recA*) were analyzed by RT-qPCR. Our results indicate that the *sod* gene plays important role in biofilm formation in *L. monocytogenes*.

**Table 1:** Strains and plasmids used in this study.

Strains and plasmids	Genotype/relevant characteristics	Source
*L.M.*4b G	Wild-type *L. monocytogenes*, serotype 4b group, isolate strain	Hubei Province Center for Disease Control and Prevention, Hubei, China
*Δ*1771	lm.G_1771 deletion mutant of *L. monocytogenes* 4b G by pKSV7::*sod*14	Laboratory stock
*Δsod*	*sod* gene deletion mutant of *L. monocytogenes* 4b G by pKSV7::*sod*14	This study
*Δ*1771*Δsod*	*sod* gene deletion mutant of *Δ*1771 by pKSV7::*sod*14	This study
DH 5α	Plasmid Host	Laboratory stock
TG1	Plasmid Host	Laboratory stock
pKSV7	Temperature-sensitive shuttle vector	Laboratory stock

## Materials and Methods

### Bacterial Strains and Growth Conditions

Strains and plasmids used in this study are listed in Table 1. Wild-type *L. monocytogenes* 4b G strain belonging to the serotype 4b group was obtained from the Hubei Province Center for Disease Control and Prevention (Hubei, China). The lm.G_1771 deletion mutant *Δ*1771 was generated from *L. monocytogenes* 4b G in the previous study [20].

**Table 2:** Primers used in this study.

Name	Oligonucleotides (Restriction enzyme sites are underlined)
*sod*1	5′-CGCGAATTCATCATCCTTATCCAGTGTTC-3′ (*Eco*R I)
*sod*2	5′-CGCGGATCCATTCCTCCTTGTATTGTTT-3′ (*Bam*H I)
*sod*3	5′-CGCGGATCCTAATCACAAGACTCACTTCGG-3′ (*Bam*H I)
*sod*4	5′-CGCGTCGACTCTTTCAAATCACGGTCAGTTA-3′ (*Sal* I)
M13F	5′-GGTTTTCCCAGTCACGAC-3′
M13R	5′-AGCGGATAACAATTTCACAC-3′
16S_F	5′-CACTGGGACTGAGACACGG-3
16S_R	5′-GGACAACGCTTGCCACCTA-3
1771_F	5′- TAAATGATACTTCCCGTTGCT -3
1771_R	5′-TTTCCCTCCTGAATCTGTGA-3
*sod*_F	5′-AAGCCCAGCCAGAACCA-3
*sod*_R	5′-GCGCTGTCCGTAACCAC-3
*kat*_F	5′- AAGCGTCATTGTTCCTAC -3′
*kat*_R	5′- GGAATAGTGAACCTTTCG -3′
*fri*_F	5′- ACTAGCAATCGGCGGAAGC -3′
*fri*_R	5′- TCGCCTTCTTTGTCAGTAAGC -3′
*sigB*_F	5′- GATGATGGATTTGAACGTGTGAA -3′
*sigB*_R	5′- CGCTCATCTAAAACAGGGAGAAC -3′
*perR*_F	5′- GAAGGAAACTTCCCTAAC -3′
*perR*_R	5′- GTGCTGCGAAATGTTCTA -3′
*recA*_F	5′- TGCGGAAGTACAAGCACAAG -3
*recA*_R	5′- GTGGTACAAGTGCTGCAACG -3

*L. monocytogenes* 4b G and its derivatives were cultured in Brain Heart Infusion (BHI) medium (BD, Franklin Lakes, NJ) or Trypticase Soy Broth (TSB) medium (BD, Franklin Lakes, NJ), while *E. coli* strains were grown in Luria-Bertani (LB) medium (Oxoid, Cambridge, United Kingdom). When required, ampicillin and chloramphenicol were added at 100 µg/ml and 10 µg/ml, respectively. The incubation temperature was 37°C for all bacterial strains used in this work, unless otherwise indicated.

**Figure 1:**
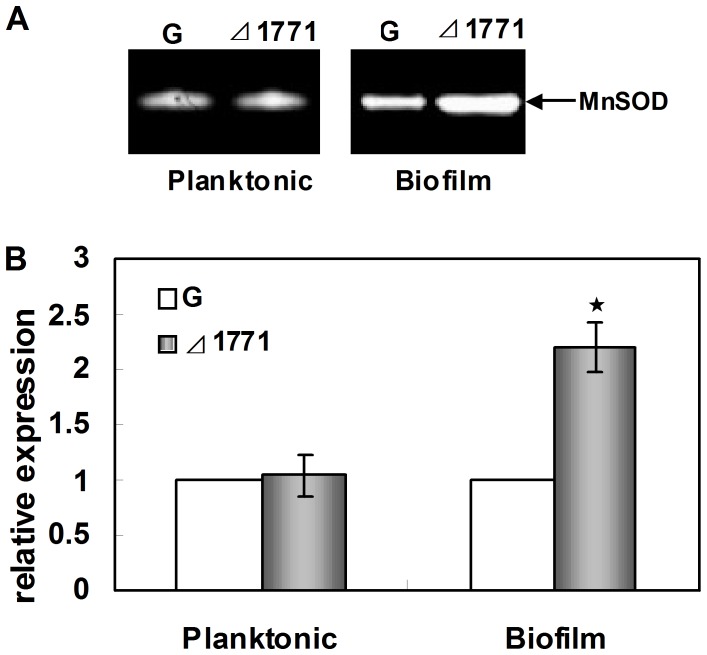
Expression of SOD in *Δ*1771 mutant. Planktonic cells were cultured in TSB with shaking (180 rpm) overnight for stationary phase. Biofilm cells were cultured in a Petri dish containing 20 ml of TSB medium and a sterilized glass slide at 37°C for 3 days. Sessile biofilm cells of both strains grown on glass slides were scraped off with cotton sticks, resuspended in distilled physiological saline, and subsequently harvested by vortexing the sticks. (A) Detection of the SOD activity in the acrylamide gels. Extracts from cells grown in planktonic and in biofilms were separated on a nondenaturing polyacrylamide gel and stained for SOD activity. (B) Transcription analysis of the *sod* gene using RT-qPCR. The transcription level of the *sod* gene in the wild-type (G) were used as the standard. The columns represent mean fold changes, and error bars show standard deviations of the means (n = 9). Star marker indicated that fold-changes were significant different (*p*-value<0.05).

### Zymographic Analysis

The SOD activities were determined by negative staining on native polyacrylamide gels [24]. Total protein of strains were prepared using the procedure of Tremoulet *et al* [16]. The protein concentration was determined by the Bio-Rad protein estimation kit with bovine serum albumin as the standard (Bio-Rad, Hercules, CA). 5 ug of total protein from each strain was loaded onto 12% (wt/vol) nondenaturing polyacrylamide gels and separated by electrophoresis in buffer lacking sodium dodecyl sulfate (SDS). After electrophoresis, the gel was soaked in 1.225 mM nitroblue tetrazolium solution for 45 min, washed with sterile distilled water, and soaked in a solution containing 0.028 mM riboflavin (Sinopharm, Shanghai, China) and 28 mM tetramethylethylene diamine (TEMED, Sigma-Aldrich, St. Louis, MO, USA) for 45 min. Subsequently, the gel was exposed to light to initiate the photochemical reaction. The SOD activity could be monitored as a clear zone surrounded by a dark blue background.

**Figure 2:**
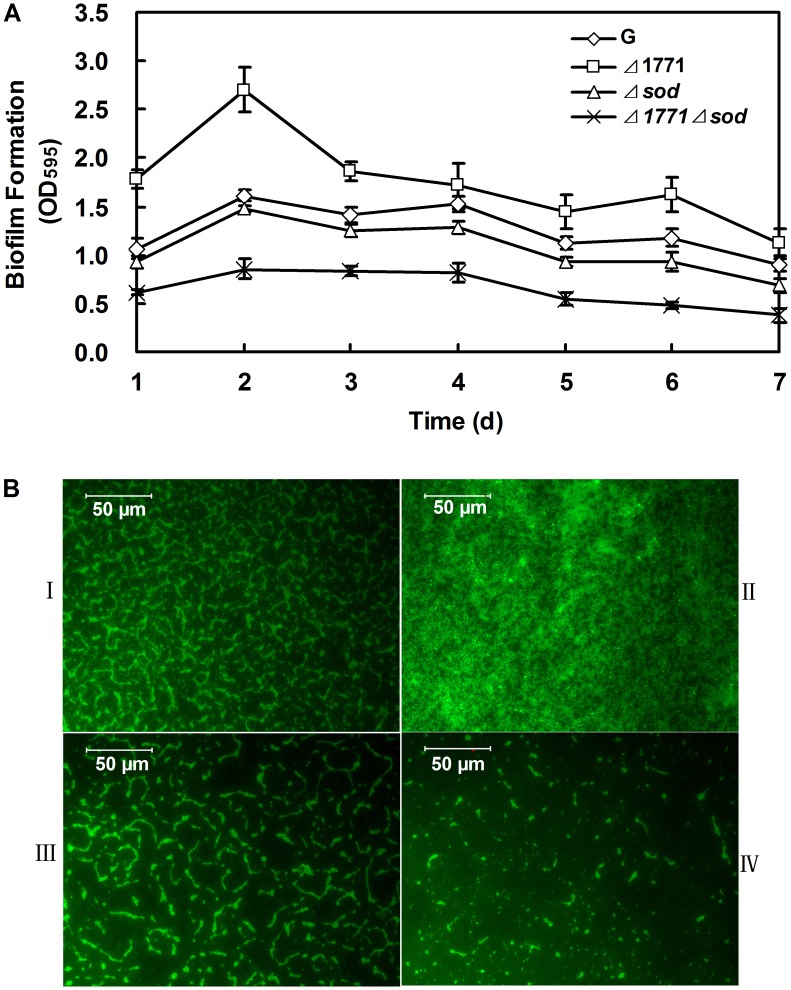
Quantification of the biofilms produced by different *L. monocytogenes* strains. (A) Microtiter plate assay: Biofilm was measured each day for cells inoculated in TSB broth with 96-well microtiter plate for 7 days. The experiments were repeated three times and error bars indicate the standard deviations. The T-test was used to calculate the *p*-value between the wild-type and mutants. (B) Microscope assay with 0.1% FITC. I: wild-type *L. monocytogenes 4b G*; II: *Δ*1771; III: *Δsod*; VI: *Δ*1771*Δsod*. Cells from each strain for this method were incubated on glass slides for 3 days to form biofilms.

### Construction of *Δsod* Mutant in *L. monocytogenes* 4b G and *Δ*1771 Mutant

Two individual in–frame deletion mutants of *L. monocytogenes* 4b G and *Δ*1771 were constructed using the vector construction technique, followed by allelic replacement [19]. This procedure relies on two recombination events: homologous recombination into the *L. monocytogenes* chromosome of a truncated copy of the gene of interest carried on the suicide shuttle vector pKSV7, followed by a second recombination event leading to the loss of the *sod* gene along with the suicide vector.

**Figure 3:**
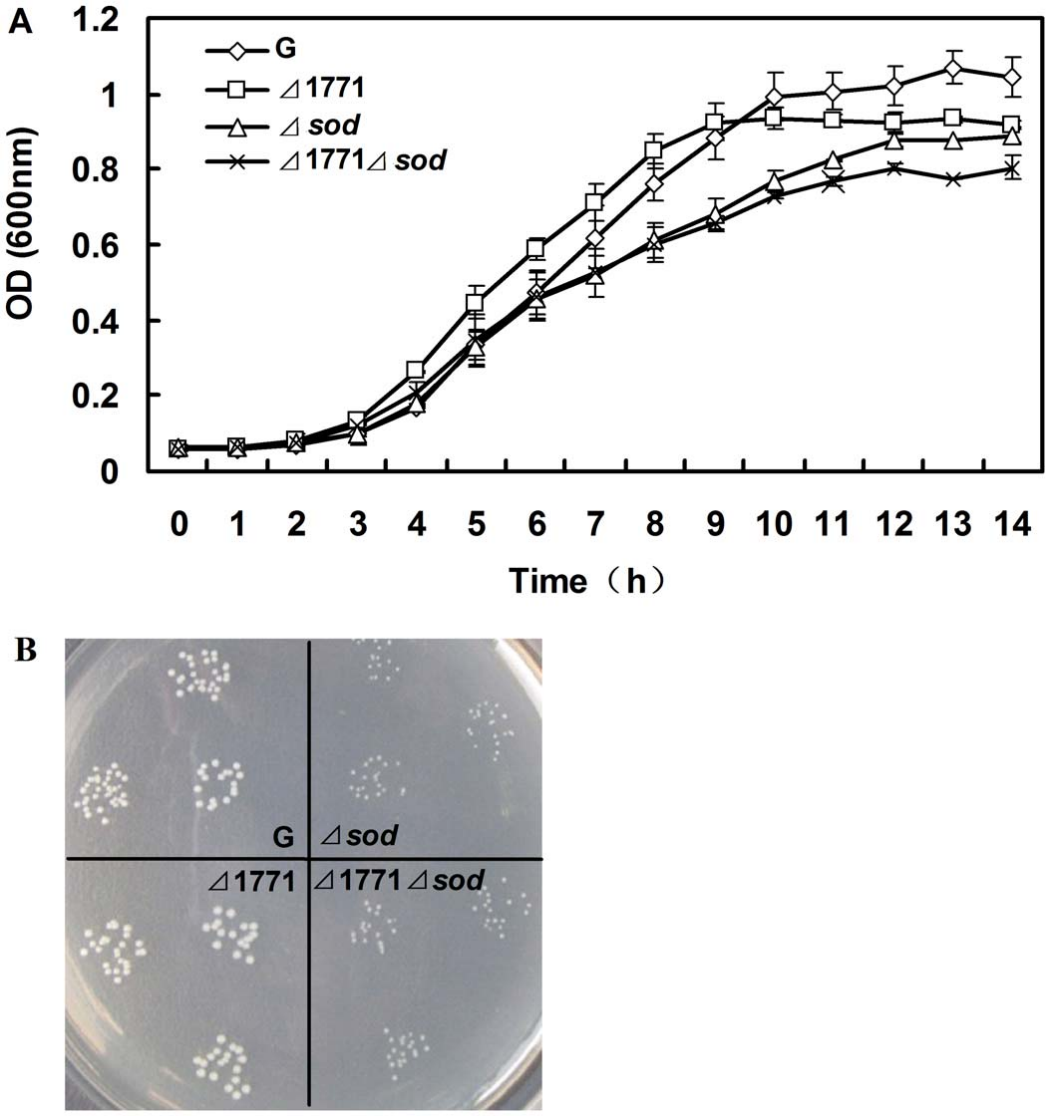
Growth studies of the different *L. monocytogenes* strains in TSB at 37°C aerobically. (A) Growth curves of different strains were measured by spectrophotometry. Mean values are expressed as the measurements of OD_600_±S.D. (n = 3). (B) Colony cultures of four strains in TSA plates for 20 h. Each strain was plated on TSA plates with triplicates.

First, for the construction of the *sod* in-frame deletion mutant, each of ∼600-bp flanking sequence at upstream and downstream of the *sod* gene was amplified by PCR from genomic DNA of *L. monocytogenes* 4b G using two primer sets *sod*1/*sod*2 and *sod*3/*sod*4 (Table 2), respectively. The upstream *Eco*R I- *Bam*H I and downstream *Bam*H I- *Sal* I fragments were cloned sequentially into the thermosensitive vector pKSV7 to generate a plasmid pKSV7::*sod*14. The recombinant plasmid was transformed into *E. coli* TG1 competent cells, and the presence of the specific in-frame deletion was verified by sequence analysis of the recombinant plasmid using a primer pair (M13-F/M13-R).

**Figure 4:**
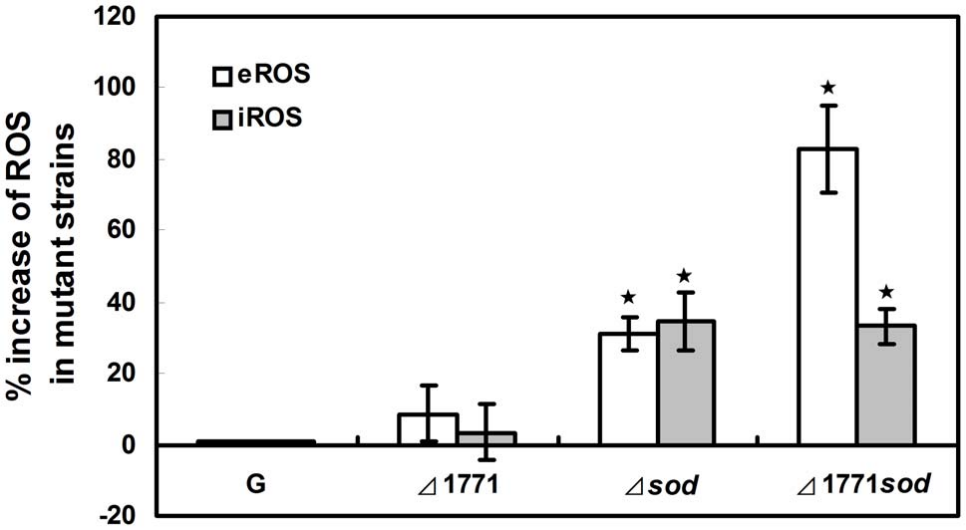
ROS assayed by chemiluminescence in planktonic cells of different *L. monocytogenes* strains. Percentages of extracellular (open bars) and intracellular (grey bars) increase of ROS generated in mutants compared to the wild type (WT) *L. monocytogenes* 4b G. The relative percentages in mutants were calculated as follows: (mutant OD_575_-WT OD_575_)/WT OD_575_. Cells were cultured in TSB with shaking (180 rpm) overnight to stationary phase, production of ROS by 10^9^ bacteria. The experiments were repeated three times and error bars indicate the standard deviations. Stars indicate that changes were significantly different (*p*-value<0.05, n = 3).

Second, the recombinant plasmid was purified and electroporated into *L. monocytogenes* 4b G and *Δ*1771, respectively. Transformants were selected by growth for 2 days at 30°C on BHI agar plates containing chloramphenicol (10 µg/ml). Subsequently, plasmid integration was forced by growing the organisms at a nonpermissive temperature (42°C) on BHI agar plates in the presence of chloramphenicol. Plasmid excision was achieved by continuous passage of cells growing at 30°C in BHI medium containing no antibiotics with shaking and spreading at intervals onto BHI agar plates. Replica plating on BHI agar plates with and without 10 µg/ml chloramphenicol allowed screening for vector loss. Homologous recombination was confirmed by PCR using outside primers *sod*1/*sod*4 (Table 2) with chloramphenicol-sensitive colonies.

**Figure 5:**
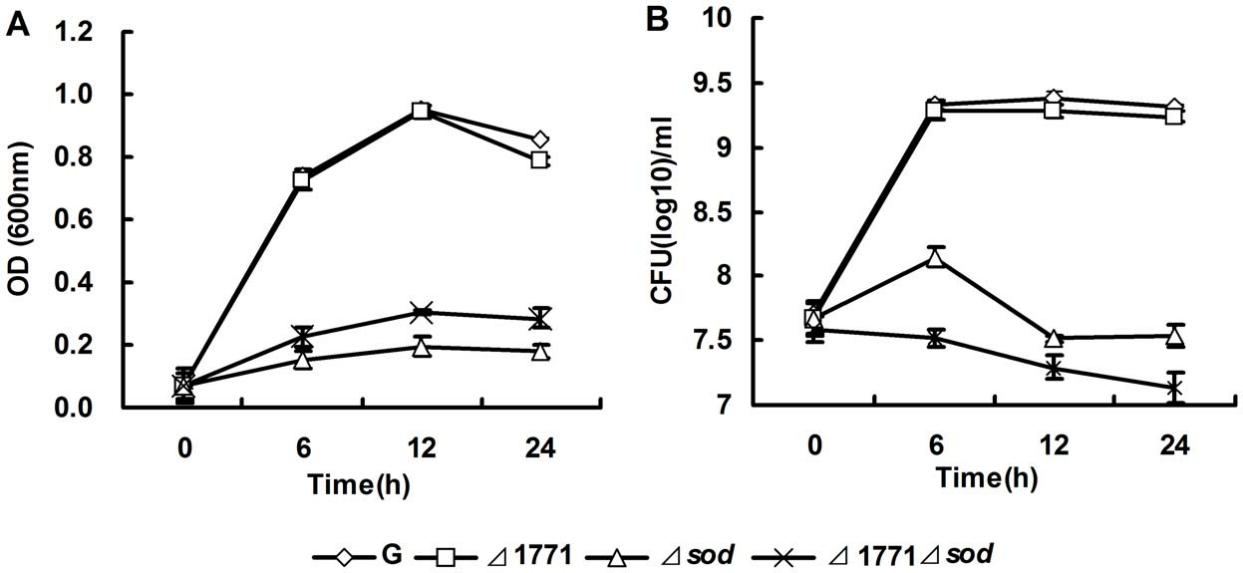
MV tolerance in planktonic cells of different *L. monocytogenes* strains. The wild-type *L. monocytogenes* 4b G (G), *Δ*1771, *Δsod* and *Δ*1771*sod* were grown in TSB +1 mM MV broth respectively, measured the absorbance at OD_600_ (A) and plated on TSB agar plates (B). The experiments were repeated three times and error bars indicate the standard errors of the mean.

### Biofilm Assay

Biofilm formation was quantified by two independent methods in this study according to Zhu *et al* [19]. First, a static biofilm formation assay was performed in 96-well polystyrene staining with 0.1% crystal violet. Each data point was averaged from at least 18 replicate wells (6 wells from each of 3 independent cultures). Second, samples were prepared on glass slides staining with 0.1% FITC (Sigma-Aldrich, St. Louis, MO, USA). The biofilms were examined with the computer-assisted Olympus fluorescence microscope BX51 (Olympus, Japan) and photographed.

**Figure 6:**
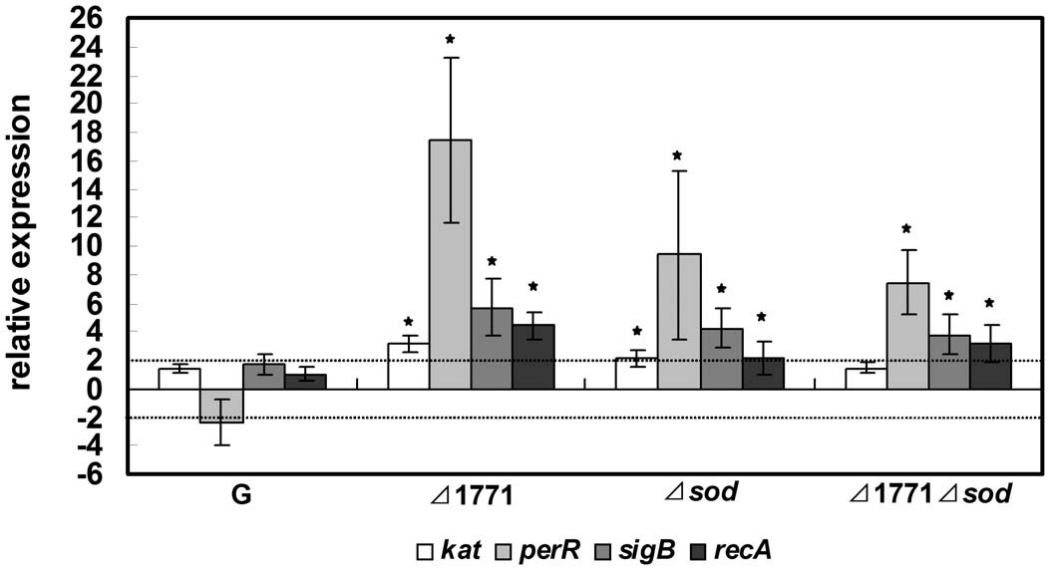
Expression of four oxidative stress-related genes in biofilms of different *L. monocytogenes* strains. 3-day planktonic cells from different strains grown statically were used as standards. Data shown are mean values with standard deviations (n≥6). Stars (★) indicate that fold-changes were significantly different (*p*-value<0.05).

### Growth Study of *L. monocytogenes* Wild-type and Mutant Strains

The wild-type *L. monocytogenes* 4b G strain and mutant strains (*Δ*1771, *Δsod* and *Δ*1771*Δsod*) were cultured overnight in 2 ml of TSB at 37°C in a shaking incubator at 180 rpm. Each culture was inoculated into 200 ml of fresh TSB at 1∶100 in one 500-ml flask and cultured at 37°C in a shaking incubator at 180 rpm for 14 h. OD_600_ was measured every hour and averaged absorbance readings were plotted against time points to produce a growth curve. All experiments were performed in triplicate.

Ten-fold serial dilutions of stationary phase cells were made for each strain. Subsequently, 10 µl from each strain with the appropriate dilutions were plated onto TSB agar medium and inoculated at 37°C for 20 h. The growth of each strain was expressed as both OD_600_ and colony size.

### ROS Determination in Planktonic Cells

The extracellular (eROS) and intracellular (iROS) production of ROS was detected by the reduction of nitro blue tetrazolium (NBT) (Sigma-Aldrich, USA) to nitro blue diformazan. Bacterial suspensions (500 µl of stationary phase) were incubated with 500 µl of NBT (1 mg/ml) at 37°C for 30 min. The reaction was stopped with 100 µl of 0.1 M HCl and the blue color of the supernatants was measured at 575 nm (eROS). Bacterial cells were separated from the supernatant by centrifugation at 1,500 g for 10 min, and then treated with 600 µl DMSO and 800 µl of PBS, pH 7.0. Reduced NBT was measured as formazan blue at 575 nm (iROS). The byproducts of the assay, which were proportional to ROS, were measured at OD_575_ [10], [11], [25].

### Methyl Viologen (MV) Treatment

The internal oxygen radical generator methyl viologen (MV, Sigma-Aldrich, St. Louis, MO, USA) was added to TSB to test the susceptibility of the wild-type and the mutants to ROS. Briefly, 0.05 ml overnight cultures were inoculated in 5 ml of fresh TSB and TSB containing 1 mM MV, respectively. Each strain was on placed in four wells and incubated at 37°C in a shaking incubator at 180 rpm for 24 h. A microplate reader (Tecan Sunrise, Switzerland) was used to measure OD_600_ at 0 h, 6 h, 12 h and 24 h, and viable colonies of each strain were counted. Growth under MV and without MV was tested separately based on the absorbance reading and colony counts at different time points.

### Transcription Analysis Using Real-time Quantitative PCR (RT-qPCR)

The bacterial pellets were resuspended in 100 µl TE buffer containing 10 mg/ml of lysozyme (Roche Diagnostics, Penzburg, Germany), subsequently incubated at 37°C for 20 min, then harvested by centrifuging at 12,000 rpm for 1 min. Total RNA was extracted with trizol reagent according to the manufacturer’s protocol (Invitrogen, Carlsbad, USA). DNase I treatment and reverse transcription were performed using the PrimeScript®RT reagent kit with gDNA Eraser (TaKaRa, Japan).

The RT-qPCR was performed on an iQ Cycler (Bio-Rad, Watford, UK). The primers were designed by express primer 3.0 software and synthesized by Sangon Co. Ltd. (China). The reaction solution was as follows: ≤1 µg Power SYBR Green PCR Master Mix (2×) (TaKaRa, Japan), 900 nM Forward Primer, 900 nM Reverse Primer, and 1–100 ng cDNA template, adding nuclease-free water up to 20 µl. The amplification program was as follows: one cycle at 95°C for 5 min, 35 cycles at 95°C for 15 s, 60°C for 15 s and 72°C for 15 s. A melting-curve analysis between 60°C and 95°C was performed after each PCR to check the specificity of the amplification product. The 16S_F and 16S_R primer set (Table 2) used to perform the real-time PCR of the 16S rRNA internal control has been previously described by Werbrouck *et al* [26].

### Data Analysis

The efficiencies of amplifications were calculated using the formula E = [10^(1/−s)^−1]×100, where “s” is the slope of the standard curve with several dilutions of cDNA. The RT-qPCR data was analyzed by the comparative critical threshold method (2^−*ΔΔ*CT^) since the application efficiencies for all the primer pairs were close to 100%. A gene is usually regarded as up- or down-expressed when its relative expression (RE) level is >2 as previously determined by Desroche *et al* [27]. The cDNAs were synthesized from RNA extracted from three independent cultures. The RE for each gene was measured in triplicate. RE was significantly different when the *p*-value was <0.05 in the T-test.

## Results

### SOD is Negatively Regulated by the ABC Transporter Permease in *L. monocytogenes* 4b G in Biofilm Formation

Our previous studies showed that the *sod* gene and protein were up-regulated in *Δ*1771 by microarray analysis and 2-D electrophoresis, respectively [20]. To confirm the relationship between the putative ABC transporter permease and SOD, the SOD activity was determined in the wild-type and mutant *Δ*1771 by zymographic assay (Figure 1). Only one active band was seen in the both strains. Clearly, inactivation of the lm.G_1771 gene induced more SOD activity in the biofilms of *Δ*1771 mutant than in the wild-type, while there was no apparent difference between the two strains in planktonic cells (Figure 1A). Our RT-qPCR analysis showed that the transcriptional level of the *sod* gene was about 2-fold up-regulated (*p*<0.05) in *Δ*1771 compared to the wild-type in biofilms, but not in planktonic cells (Figure 1B). In addition, RT-qPCR analysis revealed that there was no apparent difference in the transcriptional level of the lm.G_1771 gene between *Δsod* and the wild-type in planktonic cells or biofilm cells (data not shown).

Taken together, our data clearly demonstrated that SOD was activated at the gene, protein and activity levels in biofilms of *Δ*1771 mutant, indicating that SOD was negatively regulated by the putative ABC transporter permease.

### SOD has a Negative Effect on Biofilm Formation in *L. monocytogenes* 4b G

To determine the role of SOD in biofilm formation and the relationship with the putative ABC transporter permease, the full-length *sod* gene fragment (609 bp) was deleted in the wild-type and *Δ*1771 mutant, respectively. *Δ*sod mutants and *Δ*1771*Δsod* double mutants were verified by PCR detection using a primer pair (*sod*1/*sod*4) (Table 2, data not shown). The in-frame deletions were confirmed by sequencing. The failure of *sod* expression in *Δsod* and *Δ*1771*Δsod* mutants were also confirmed by RT-qPCR and SOD activity assays (data not shown).

The biofilm formation of the wild-type *L. monocytogenes* 4b G and the deletion mutants (*Δ*1771, *Δsod* and *Δ*1771*Δsod*) were examined using microtiter plate method and microscope assay, respectively (Figure 2). As shown in Figure 2A, *Δ*1771 formed more biofilm compared to the wild-type during 7 days, while both *Δsod* and *Δ*1771*Δsod* mutants formed less biofilm than the wild-type (*p*<0.01). These results were also confirmed by microscopic assay (Figure 2B). Our results indicate that SOD negatively regulates biofilm formation in *L. monocytogenes* 4b G.

### SOD Influences the Growth of *L. monocytogenes* 4b G and Contributes to the Survival of Cells in Oxidative Stress

To analyze the influence of the putative ABC transporter permease and SOD on bacterial growth, the growth rates of the wild-type *L. monocytogenes* 4b G, *Δ*1771, *Δsod* and *Δ*1771*Δsod* mutants were monitored. Both *Δsod* and *Δ*1771*Δsod* mutants grew slower than the wild-type strain in liquid TSB medium, while there was no distinct variation in the growth rate between *Δ*1771 and the wild-type (*p*>0.05) (Figure 3A). Moreover, the colony sizes of *Δsod* mutant were about half of the wild-type on solid TSB medium (Figure 3B). This indicates that SOD was responsible for the slow growth of *L. monocytogenes* 4b G on TSB under aerobic conditions.

The production of ROS was measured in the wild-type *L. monocytogenes* 4b G, *Δ*1771, *Δsod* and *Δ*1771*Δsod* mutants (Figure 4). Both extracellular and intracellular production of ROS (eROS and iROS) was increased in *Δsod* and *Δ*1771*Δsod* mutants compared to the wild-type (Figure 4). However, the influence of the putative ABC transporter permease on ROS production was not significant (Figure 4).

MV is an internal oxygen radical generator that increases the level of ROS in the cell [28], [29]. The sensitivities of putative ABC transporter permease and SOD to MV were monitored (Figure 5). In the presence of MV, both *Δsod* and *Δ*1771*Δsod* mutants showed reduced viability compared to the wild-type and *Δ*1771 mutant (Figure 5A). Similar results were obtained using colony count methods (Figure 5B). Our results indicate that mutant cells had more internal ROS; therefore, adding more ROS through MV is detrimental to cells.

### Four Oxidative Stress-related Genes were Induced in Biofilm of *Δsod* Mutant

The biofilm formation of the wild-type was greatly reduced after 1 mM MV treatment indicating that excess ROS inhibits biofilm formation (data not shown). The fact that our mutants had more internal ROS (Figure 4) may be responsible for their reduced levels of biofilm formation. To test this hypothesis, four genes (*kat*, *perR*, *sigB* and *recA*) related to ROS were selected to study their roles in biofilm formation in the mutants. Transcription analysis of these four genes was performed between biofilm and planktonic cells in the four strains (*L. monocytogenes* 4b G, *Δ*1771, *Δsod* and *Δ*1771*Δsod*) using RT-qPCR (Figure 6). Both biofilm and planktonic cells for RT-qPCR were cultured under the same growth conditions. As shown in Figure 6, all four genes were up-regulated in biofilm cells of the deletion mutants compared to the wild-type. The elevated levels of oxidative stress-related genes in the mutants may contribute to decrease the ROS in *Δsod* mutants.

## Discussion

Our previous work identified that the *sod* gene and protein were up-regulated in the *Δ*1771 mutant [20]. In the present work, we found that the SOD activity was induced specifically in the biofilm of *Δ*1771 mutant. We also found that the *Δsod* mutant displayed a slow growth phenotype, deficient in biofilm formation, produced more ROS and sensitive to MV treatment compared to the wild- type. Moreover, four genes related to oxidative stress were also up-regulated in the biofilms of *Δsod* mutant. Based on our previous work, we hypothesized that lm.G_1771 negatively regulated biofilm formation through the expression of *sod*. The high expression of SOD in *Δ*1771 mutant may help biofilm formation because the induction of *sod* was only present in biofilm cells and not in planktonic cells. Since oxidative stress influences biofilm formation, the denser biofilms in *Δ*1771 may produce more oxidative stress than the wild-type, indicating that the up-regulation of the *sod* gene is needed to cope with oxidative stress, as described by Tremoulet *et al* [16]. This hypothesis was confirmed by the fact that the *Δsod* mutant produced more ROS than the wild-type, and thereby exhibited the reduced ability in biofilm formation. The *Δsod* mutant is also more sensitive to MV due to its high level of internal ROS in planktonic cells. Genes related to ROS were also up-regulated in biofilm formation. Taken together, our results suggest that the *sod* gene plays an important role in biofilm formation in *L. monocytogenes* 4b G.

Biofilm formation involves a complex regulatory network [30]. Moreover, biofilm can produce oxidative stress to cells and influences the expression of other genes such as anti-oxidative genes (*kat*), stress regulators (*perR* and *sigB*) and the DNA repair gene (*recA*) besides the *sod* gene. *Kat* is an important anti-oxidative gene encoding catalase that catalyzes the disproportionation of H_2_O_2_ to oxygen and water, usually working synergistically with *sod* [31]. In this study, the up-regulated expression of this gene in biofilm cells indicates that oxidative stress indeed exists in biofilm formation. Some regulators, such as *perR* and *sigB*, are also involved in anti-oxidative stress. *PerR* regulates a number of genes that play a critical role in the defense against peroxide stress and ROS [32]. *SigB* encoding a major stress response regulator has a central role in response to various stress conditions as an autoregulatory alternative sigma factor [17], [33]. In *Bacillus subtilis*, the *perR*-dependent specific stress response and the *sigB*-dependent general stress act together to make cells more resistant to oxidative stress [34]. Therefore, the higher expression of *perR* and *sigB* in biofilm formation suggests that biofilm formation stimulates the expression of the two regulators against stress when the antioxidant defenses are deficient.

Redundant ROS are known as triggers of SOS response that are involved in DNA repair [35]. *RecA* is required for SOS activation, homologous recombination and DNA repair [36], [37]. In this study, the high level of ROS (eROS and iROS) output (Figure 4) and the up-regulation of *recA* (Figure 6) in mutant strains may imply that the SOS response was induced to cope with damage from ROS in mutants. Van der Veen *et al*. [18] found that the *recA* gene was required for activation of the SOS response against damage to cells during continuous-flow biofilm formation. The increased expression of *recA* in biofilm cells of the three mutants, but not in the wild-type strain, may indicate that the putative ABC transporter and SOD play important roles in maintaining normal cells growth in biofilm formation.

In conclusion, our results demonstrated that SOD, which is negatively regulated by the putative ABC transporter permease, may be required for the biofilm formation in *L. monocytogenes* 4b G. *Sod* and 1m.G_1771 contribute to the maintenance of normal cells in biofilm formation of *L. monocytogenes* 4b G. Moreover, anti-oxidative genes, stress regulators, and DNA repair genes were necessary in deficient antioxidant defense strains to form biofilms. Further research on the relationship between biofilm formation and oxidative stress will enhance our understanding about the mechanism of bacterial survival in biofilms.
